# Methodological Improvements With Conductive Materials for Volume Imaging of Neural Circuits by Electron Microscopy

**DOI:** 10.3389/fncir.2018.00108

**Published:** 2018-11-23

**Authors:** Huy Bang Nguyen, Truc Quynh Thai, Yang Sui, Morio Azuma, Ken Fujiwara, Nobuhiko Ohno

**Affiliations:** ^1^Division of Neurobiology and Bioinformatics, National Institute for Physiological Sciences (NIPS), Okazaki, Japan; ^2^Department of Anatomy and Structural Biology, Interdisciplinary Graduate School of Medicine and Engineering, University of Yamanashi, Chuo, Japan; ^3^Department of Anatomy, Faculty of Medicine, University of Medicine and Pharmacy (UMP), Ho Chi Minh City, Vietnam; ^4^Department of Anatomy, Division of Histology and Cell Biology, School of Medicine, Jichi Medical University, Shimotsuke, Japan

**Keywords:** scanning electron microscopy, volume imaging, charging, ketjen black, conductive resin

## Abstract

Recent advancements in electron microscope volume imaging, such as serial imaging using scanning electron microscopy (SEM), have facilitated the acquisition of three-dimensional ultrastructural information of biological samples. These advancements help build a comprehensive understanding of the functional structures in entire organelles, cells, organs and organisms, including large-scale wiring maps of neural circuitry in various species. Advanced volume imaging of biological specimens has often been limited by artifacts and insufficient contrast, which are partly caused by problems in staining, serial sectioning and electron beam irradiation. To address these issues, methods of sample preparation have been modified and improved in order to achieve better resolution and higher signal-to-noise ratios (SNRs) in large tissue volumes. These improvements include the development of new embedding media for electron microscope imaging that have desirable physical properties such as less deformation in the electron beam and higher stability for sectioning. The optimization of embedding media involves multiple resins and filler materials including biological tissues, metallic particles and conductive carbon black. These materials alter the physical properties of the embedding media, such as conductivity, which reduces specimen charge, ameliorates damage to sections, reduces image deformation and results in better ultrastructural data. These improvements and further studies to improve electron microscope volume imaging methods provide options for better scale, quality and throughput in the three-dimensional ultrastructural analyses of biological samples. These efforts will enable a deeper understanding of neuronal circuitry and the structural foundation of basic and higher brain functions.

## Introduction

The brain is composed of circuits of neurons connected to one another by neurite projections, which enables information processing in the nervous system. Impairment of neural circuitry is associated with psychiatric and neurological disorders, and a complete understanding of the wiring diagram of neuronal connections, termed the “connectome,” will provide important clues to understand brain functions and develop treatments for psychiatric and neurological disorders (Filippi et al., 2013; Deco and Kringelbach, 2014; Fornito et al., 2015). To completely understand neural circuitry, multiple imaging approaches are needed to analyze various brain structures (Le Bihan et al., 2001; Fenno et al., 2011; Grienberger and Konnerth, 2012; Lichtman et al., 2014; Ohno et al., 2016). Light microscopic technologies have enabled high-throughput and detailed analyses of neuronal circuits at a very large scale (Wilt et al., 2009; Osten and Margrie, 2013). In addition, the development of cell-specific labeling with genetically encoded tags led to marking of brain cells with different colors and tracking of specific neuronal projections at the whole-brain level (Gong et al., 2003; Livet et al., 2007). Studies on such “mesoscopic connectome” achieved big datasets and demonstrated the physical and functional connections among neurons which can span the whole brain, but a deeper understanding on neuronal circuitry has been hampered by several factors (Ohno et al., 2016). Among them, one critical factor of light microscopic approaches is the difficulty to ensure synaptic connections of fine projections, because the resolution of light microscopy is limited. The processes of neurons can be ~50 nm in diameter, and the neck of the dendritic spines can be even thinner (Briggman and Bock, 2012). These structures are too small to resolve with light microscopes for volume imaging of the brain. To overcome this problem, the standard approach is electron microscopic observation at the level of individual synapses, which unequivocally visualize fine projections and physical connections among neurons through synapses using serial section images at the ultrastructural level (Palay, 1958; Brightman and Reese, 1969). Serial electron microscope images and reconstruction of three-dimensional ultrastructural information are powerful approaches to understand the neuronal connectivity of complex brain architectures.

The three-dimensional reconstruction of biological samples has been made possible using serial ultrathin sections observed by scanning (SEM) or transmission electron microscopy (TEM; Harris et al., 2006; Bock et al., 2011; Briggman and Bock, 2012). The throughput of these microscopy techniques has recently increased significantly (Briggman and Bock, 2012). In the case of SEM, new section collection procedures such as focused ion beam SEM (FIB-SEM; Knott et al., 2008), serial block-face SEM (SBEM or SBF-SEM; Denk and Horstmann, 2004) and automated tape-collecting ultramicrotome (ATUM; Hayworth et al., 2014) are revolutionizing the field of volume electron microscopy. These new TEM- and SEM-based approaches are often complementary and differ in resolution, throughput, sample types and post-acquisition image alignment. In this context, the SEM-based methods have recently advanced our understanding of three-dimensional structures in various organelles, cells, tissues and organisms in life science and clinical medicine, including large scale neural wiring maps of various organisms (Briggman et al., 2011; Kubota et al., 2011; Holcomb et al., 2013; Terasaki et al., 2013; Ohno et al., 2014; Ichimura et al., 2015; Kasthuri et al., 2015; Katoh et al., 2017). In addition, new devices to image large tissue areas, such as multi-beam SEM, have been developed and facilitated data acquisition from very large tissues such as whole brains (Eberle et al., 2015).

At the same time, methods using SEM for serial image acquisition generally require specific sample preparation techniques, in particular for the acquisition of large stacks of serial images with satisfactory contrast for subsequent tissue annotation, segmentation and analysis. For example in SEM imaging, the available parameter range for beam irradiation, e.g., beam current and voltage, is limited by insufficient conductivity of the biological samples. In order to acquire high contrast and high quality images, it is preferable to have sufficient deposition of heavy metals in the sample. To overcome these problems, extensive efforts have been made to improve throughput and image quality from SEM-based imaging in large tissue volumes.

Here, we review recent methodological advances in volume imaging using SEM with particular emphasis on newly developed approaches and conductive materials used in sample preparations and tissue embedding for serial sectioning and imaging, which will contribute to our understanding of the connectome in different organisms.

## Basic Methodology of Sample Preparations and Data Acquisition for Volume Imaging Using SEM

In SEM, images are produced by focusing electron beams, scanning over the bulk specimens and detecting ultrastructural information of the specimen surface using secondary or backscattered electrons (BSE). But when BSE and/or secondary electrons derived from the flat block/section surface of resin-embedded tissue samples are detected in SEM, images which are similar to those obtained from the embedded samples in TEM can be acquired (Richards and Gwynn, 1995; Wergin et al., 1997). When low electron energies are used for the block/section face imaging with SEM, the BSE contain information only from near the surface of the embedded samples (Hennig and Denk, 2007), which can result in a depth resolution of <30 nm depending on the energy of landing electrons (Denk and Horstmann, 2004; Knott et al., 2008). For these reasons, observation of block/section faces in SEM facilitated serial image acquisition for large volume 3D reconstruction of the fine processes and synaptic connections of the nervous system, but requires specific sample preparation which can be distinct from conventional approaches for TEM or SEM observation.

**Figure 1:**
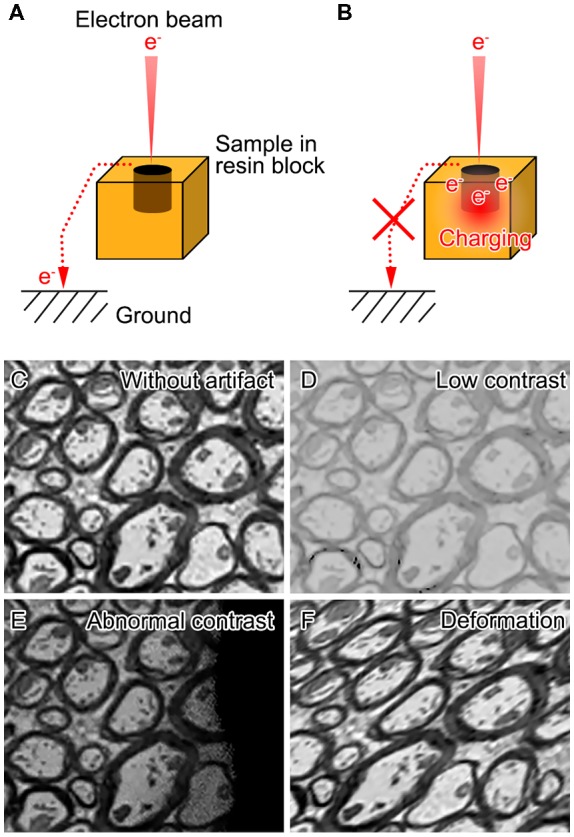
Charging in scanning electron microscopic imaging and artifacts caused during the acquisition of block-face images with scanning electron microscopy (SEM). When the specimens (resin blocks with samples) are sufficiently conductive **(A)**, excessive electrons in the incident electron beam reach the ground. When the conductivity of the specimens is low **(B)**, some of the incident electrons accumulate on the surface of the specimens and cause charging. Schematic images show that the artifacts that can be observed in the block-face image of the mouse spinal cord **(C)** involve low contrast **(D)**, or abnormal contrast **(E)** and deformation **(F)** due to sample charging.

**Figure 2:**
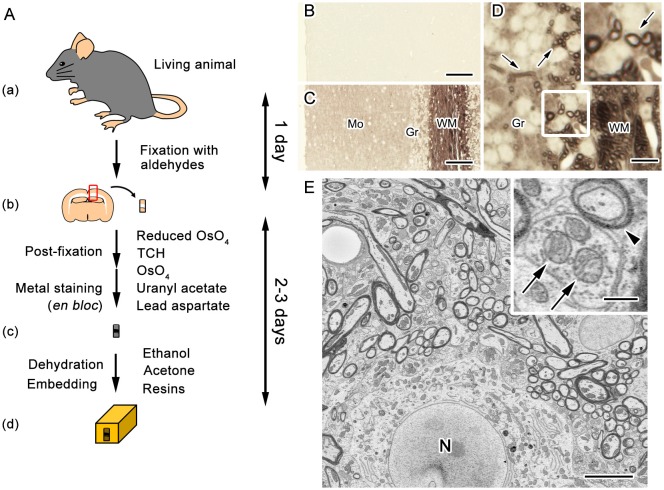
*En bloc* staining with dense heavy metal deposition facilitates image acquisition with SEM. A diagram of the procedure for sample preparation widely used in serial block-face (SBF) imaging with SEM **(A)**. Fixation of target tissues (mouse brain in this case) is performed by the common perfusion or immersion fixation using aldehyde fixatives (a,b). Post-fixation along with *en bloc* staining with metals is performed through treatments with ferrocyanide-reduced osmium tetroxide (OsO_4_), thiocarbohydrazide (TCH), OsO_4_, uranyl acetate and lead aspartate (b,c). The specimens are embedded after staining in epoxy resins following dehydration with organic solvent (c,d). Light microscope images of unstained sections obtained from cerebellar tissues embedded in epoxy resin **(B–D)**. The sections were prepared with either the standard procedure for transmission electron microscopy (TEM) including only post-fixation with OsO_4_ **(B)**, or the procedure for volume imaging, which includes treatments with reduced OsO_4_, thiocarbohydrazide, OsO_4_, uranyl acetate and lead aspartate **(C,D)**. Compared with the standard procedure for TEM **(B)**, the procedure for volume imaging clearly visualized histological features **(C)**, such as myelinated nerve fibers (**D**, arrows). Mo, molecular layer; Gr, granular layer; WM, white matter. For SEM imaging, cellular structures, such as myelin membranes (**E**, arrowhead) and mitochondria (**E**, arrows), were clearly observed in samples with dense heavy metal staining. N, nucleus. Bars: 50 μm **(B,C)**, 12.5 μm **(D)**, 5 μm **(E)** or 500 nm (**E**, inset). Images were adapted from Ohno et al. (2015) with permission.

Biological samples are mostly composed of light elements such as carbon, oxygen, hydrogen and nitrogen, and therefore imaging non-conductive biological specimens with SEM is often hampered by artifacts associated with charging and insufficient contrast (Figure 1). Various efforts have been made to achieve higher contrast and better resolution for volume imaging of biological specimens under SEM. These efforts consist of modifications of different steps including post-fixation, staining, embedding and image acquisition (Figure 2A).

Most tissue preparation procedures for serial imaging with SEM include common fixation with chemicals such as aldehydes and *en bloc* metal staining involving osmium, uranium and lead. Following these post-fixation and staining procedures, the small pieces of tissue blocks are embedded in common resins. Efficient acquisition and analyses of serial electron microscope images are facilitated by higher contrast in cells and organelles, and therefore the procedures are designed to achieve enhanced deposition and *en bloc* staining of metals, and are now widely used to observe membranous organelles and cellular morphology (Figure 2; Deerinck et al., 2010; Tapia et al., 2012; Ohno et al., 2015; Yin et al., 2016). The *en bloc* preparation is essential for block-face imaging such as SBEM and FIB-SEM, since the block-face is imaged immediately after exposure. The *en bloc* staining is also used for imaging of the sections in ATUM or TEM because of the benefits of relatively even staining and more metal deposition for increased conductivity, which results in improved contrast. As a consequence, lower beam doses can be used for imaging which reduces radiation damage. The methods to enhance membrane contrast used heavy metal deposition to cellular membranes (Seligman et al., 1966; Karnovsky, 1971; Walton, 1979). These methods have drawbacks, such as areas with limited staining and tissue destruction from the generation of nitrogen gas. Inhibition of nitrogen bubble formation along with staining of much wider areas was achieved in a method termed BROPA using the additional solvent and pyrogallol (Mikula and Denk, 2015). In addition, another method employed sequential modification of common preparation procedures to facilitate homogeneous metal deposition (Hua et al., 2015). These methods addressed the problems of stain penetration depth by modifying sample preparation methods for observation of large areas in brain tissues (Hua et al., 2015; Mikula and Denk, 2015). Collectively, these approaches including alternative reagents and devices which are combined with historical methods became powerful options for efficient acquisition of high quality datasets from various types of specimens including large brain tissues.

The development of improved staining procedures has been accompanied by the development of new in-chamber techniques for charge compensation that modify the acquisition condition inside of the SEM chambers. The next section introduces some of such mechanical improvements, which are termed “In-Chamber Techniques for Charge Compensation” in this review.

## In-Chamber Techniques for Charge Compensation

Multiple approaches have been proposed which can modify the circumstances or samples in SEM chambers in order to reduce artifacts and acquire data with higher quality. For example, observation with SEM under low vacuum conditions, such as variable-pressure SEM, has often been used to acquire images from samples with problems of charging. However, these observation methods generally involve electron-gas interactions and electron beam scattering and can reduce the signal-to-noise ratio (SNR) and worsen image quality (Mathieu, 1999). To overcome the observation problems in low vacuum conditions, focal gas injection onto the block-face was used for SBEM imaging, which was termed focal charge compensation (FCC) system (Deerinck et al., 2017). This approach substantially improved charging and enabled image acquisition from samples prepared without dense heavy-metal staining. In FCC, a retractable application nozzle, mechanically coupled to the reciprocating action of the built-in ultramicrotome, was paired with a gas injection valve. The system enables the application of nitrogen gas precisely over the block-face during imaging while the high vacuum of the specimen chamber is maintained. The locally applied nitrogen gas molecules are ionized, approach the sample surface, and neutralize electrons, which charges the sample surface (Thiel et al., 1997). As a result, the FCC system does not interfere with the operation of the SBEM, but greatly reduces image artifacts in the stacks of charge-prone specimens. The addition of FCC does not affect the total time of data acquisition, but can reduce the time by allowing shorter dwelling times due to the improved SNR. Quantitatively, when increasing the accelerating voltage from 2.5 keV to 4.0 keV (60%) and increasing the pixel dwell time from 1 μs to 4 μs (4×), SNR was 28% lower using variable pressure-SEM than FCC, and the resolution obtainable by FCC was nearly the same as measured using high vacuum (Deerinck et al., 2017). Taken together, FCC is a promising approach to observe charging-prone samples by modifying SBEM system but not samples themselves.

In addition to alterations of the sample atmosphere, beam deceleration can significantly improve the contrast and resolution of images in block-face imaging of biological samples in SEM under low landing energy levels and a low beam current (Ohta et al., 2012; Titze and Denk, 2013). In the beam deceleration approach, the specimens are held at a negative bias voltage, and the electrons leaving the column are decelerated before reaching the specimens. The beam deceleration system has multiple advantages including improved detection of signals from negatively biased specimens and better resolution by very low landing energy of the incident electrons. Although the sample conductivity is critical for the beneficial effects of beam deceleration, imaging of such conductive samples at high spatial resolution could be significantly facilitated by applying beam deceleration upon imaging in SEM.

Treatments to increase the surface conductivity of samples have been widely used in observation of biological specimens in SEM. Attempts to apply this concept to the SBEM imaging have been made in SEM chambers by automated block-face metal coating, and charging could be significantly improved during SBEM imaging (Titze and Denk, 2013). In this study, the surface of the imaged blocks was covered with thin (1–2 nm) metallic films composed of chromium or palladium using an electron beam evaporator that is integrated into the microscope chamber. In this system, the conductivity of the surface was increased by the thin metallic films prior to each cycle of imaging. The reduction in SNR caused by the metallic film is smaller than that caused by the widely used low-vacuum method. So the film coating results in better signal than the low-vacuum method, but still fully compensates any charging artifacts. In addition, one big advantage of this in-chamber coating method is that it allows detection of secondary electrons, which in turn enables much higher acquisition speeds than BSE-based imaging. The sample whose surface was 12 mm across could be coated and imaged without charging effects at beam currents of 25 nA, and more than 1,000 serial images could be acquired under the automated cut/coat/image cycles. However, one critical drawback of this approach is the requirements for the specific devices which enable in-chamber coating of the samples with the metallic films.

Another method using plasma etching prior to imaging has been used to remove contaminants and enhance contrast in serial image acquisition using ATUM (Morgan et al., 2016). Plasma cleaning has been used to remove contaminants, and this would be helpful in ATUM since it is possible that various contaminants which perturb image acquisition can be attached on the surface of sections during sectioning, mounting on the tape and subsequent preparation for imaging. In addition, plasma etching can be used to enhance contrast in SEM imaging using secondary electrons, presumably due to the removal of specimen components near the specimen surfaces and generation of surface unflatness (Hukui, 1996). The plasma etching could be beneficial in serial image acquisition in SEM when the secondary electrons are used for imaging of the block/section faces.

The modifications of physical properties, such as sample conductivity, and improvements in observation methods have improved image quality. Dense deposition of heavy metals on specimens is beneficial for SEM imaging because it increases conductivity and improves the SNR of samples. Increasing the conductivity of the embedding media in addition to specimen conductivity could be beneficial for the observation of non-conductive biological materials. Different materials and methods for specimen embedding have improved in the life sciences and clinical medicine, and in the next section we discuss several recent studies that modified embedding procedures and media in order to facilitate serial image acquisition using SEM.

## Improvement of Embedding Methods for Charging Compensation

Developing new embedding media for electron microscope imaging requires consideration of the physical properties associated with the imaging procedures, such as stability in sectioning and the degree of deformation under electron beam irradiation. Sectioning with a diamond knife requires careful consideration of the physical properties of the target materials, which significantly affect knife lifetimes (Hashimoto et al., 2016). Imaging and cutting conditions, such as sample temperature, cutting speed, cutting thickness and size, knife shape and knife temperature, also affects knife lifetimes, but material hardness is the most important factor for image quality. In addition, electron beam irradiation causes thermal damage to the resin, and artifacts occur from resin shrinkage and deformation, which can be ameliorated by cooling the samples to cryotemperatures (Luther, 2006). These artifacts also depend on electron beam properties, such as acceleration voltage strength and electron current, which can be evaluated with sections mounted on conductive tapes (Kubota et al., 2018). However, damage and structural deformation of the resin-embedded samples from electron beam irradiation may also be affected by the properties of the stained and embedded tissues.

Historically, various resins have been used for electron microscope observation of biological specimens. Early resins, such as methacrylates, developed for ultrathin sectioning and epoxy resins developed later resulted in less structural changes (e.g., shrinkage) upon curing and high stability during ultrathin sectioning and electron beam irradiation (Glauert and Glauert, 1958; Luft, 1961). Different types of resins, including water soluble and hydrophilic resins, have been developed and used for electron microscope observation, and these resins have unique properties which are suitable for different target samples and staining and observation methods (Staeubli, 1963; Leduc and Bernhard, 1967). The artifacts from shrinkage and deformation typically include depth-direction and planar shrinkage during electron beam irradiation. This type of shrinkage is obvious during SBEM imaging, when there is local failure of physical slicing in areas with intensive irradiation of the electron beam for focusing. TEM-based evaluation revealed that the stability against electron beam artifacts varies among different resins (Kizilyaprak et al., 2015). Interestingly, maximal resistance against electron beam damage is achieved by a mixture of different resins, but the exact mechanisms of improved resistance remain unclear. These studies provide options for the optimization of embedding media, which enables better stability for the imaging of biological specimens with intensive beam irradiation.

**Figure 3:**
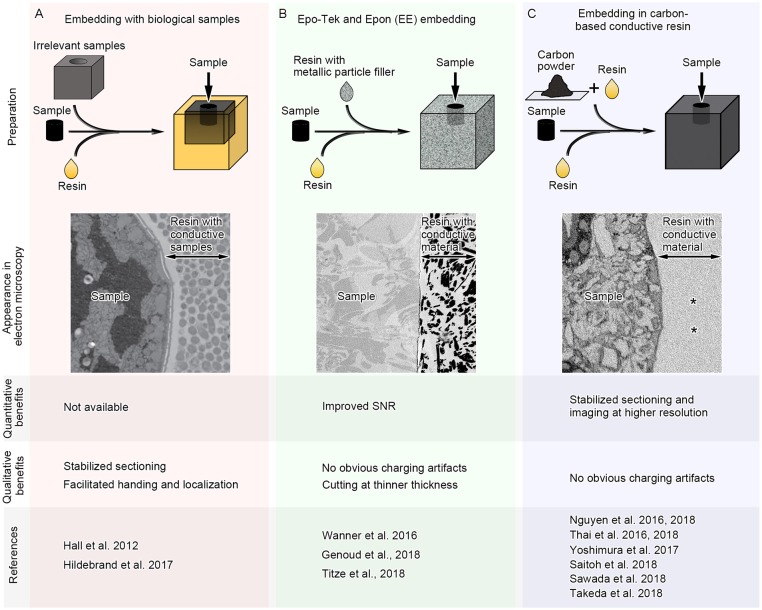
Three different approaches for serial image acquisition in SEM which modulate physical properties of resin around samples. In the first approach **(A)**, samples are embedded with irrelevant biological samples which are prepared similarly as the target samples. In the second approach **(B)**, the samples are incubated in pure resin and then embedded in conductive resin containing metallic particles. In the third approach **(C)**, the samples are incubated in pure resin and then embedded in conductive resin which is mixture of the resin and carbon black. In addition to the schemes showing preparation methods, the schematic images of the sample appearance in electron microscopy, benefits which have been quantitatively or qualitatively evaluated and references using each approach are shown. SNR, signal-to-noise ratio. Carbon-based conductive resin generates little contrast in block-face images of SEM (**C**, asterisks).

Generally, resins used for electron microscope observation have distinct physical properties compared with adjacent embedded biological specimens. Most resins are composed of light elements, which have lower conductivity than that of the embedded specimens, particularly when the specimens are densely stained with heavy metals. In addition, the hardness of the resin is altered in regions with biological specimens. These problems could be potentially solved by modifying the undesired physical properties of the resins around the samples. “Fillers” have long been used to modify the physical properties of base materials (e.g., plastics, concrete), such as electrical conductivity and hardness. It is therefore possible that those conventional or new filler materials have beneficial effects on physical properties of the resins and facilitate serial image acquisition in SEM by reducing artifacts. Recent studies have started exploring this possibility and found some promising results using different types of “fillers” beneficial for the serial image acquisition in SEM (Figure 3).

To facilitate SEM imaging, biological specimens that are not related to the experiment are embedded with the target samples. These biological specimens are used as a kind of “filler material,” which modifies the physical properties of the surrounding resin. Biological filler materials are stained and prepared similarly to the target tissues, and therefore the physical properties of the filler and target tissues are similar. One example of the biological filler materials is tissue from the mouse brain, which was embedded with larval zebrafish for serial sectioning by ATUM (Figure 3A; Hildebrand et al., 2017). Homogenous hardness and stability of sample blocks facilitate repeated serial sectioning by ATUM and prevent heterogeneous shrinkage, deformation and folding of sections. Small larval zebrafish samples were post-fixed, stained *en bloc*, embedded into resin blocks, and finally surrounded by mouse brain tissues for stabilization during sectioning. In this study, 17,963 sections at 60 nm thickness were acquired in ATUM for serial image acquisition. In total, 244 (1.34%) sections were lost and 283 (1.55%) were partially lost, while no two adjacent sections were lost. Although more quantitative analyses on physical properties of the brain “filler materials” are required, it is possible that filler biological samples treated and embedded similarly to the target specimens substantially improve production of and imaging from serial ultrathin sections.

Aggregated unicellular organisms can also be used as biological support materials. *C. elegans* was embedded with *E. coli* or yeast cells during cryofixation to facilitate handling and localization of the samples (Figure 3A; Möller-Reichert et al., 2003; Hall et al., 2012). The samples were still surrounded by biological material during the subsequent tissue preparation procedures, including freeze-substitution, and the resins surrounding the *C. elegans* sample at the time of observation were filled with biological material that was stained and embedded at the same time (Hall et al., 2012). Because the sample has biological components enriched with metal deposition, the regions occupied by the organism had different physical properties from bare resin. These types of approaches using biological filler materials are promising options to facilitate serial image acquisition using SEM.

Besides biological tissues, other filler materials have been used to modulate the physical properties of resins. For example, the addition of metal particles alters properties such as the electrical and thermal conductivity of plastic (Bhattacharya and Chaklader, 2006). Metallic particles are embedded to image brain tissues by SBEM, where samples with low metal deposition or areas of non-conductive embedding media outside of tissues are susceptible to charging artifacts (Figure 3B). Epo-Tek and Epon embedding (EE embedding) uses commercially available epoxy glue containing silver particles, and this technique enables embedding conductive resins with metal particles in the vicinity of target brain samples (Wanner et al., 2016). Although areas with less heavy metal deposition have charging artifacts, this approach facilitated serial imaging of brain samples under high-vacuum conditions. Serial images of 4,750 sections at 25 nm thickness could be acquired in this study, and only one section was lost, proving that EE-embedding is a promising approach and considered to be suitable for ultra-thin sectioning. Recent studies used the same approach, and one of them acquired 11,416 slices of tiled images at 10 nm × 10 nm × 25 nm resolution in SBEM (Genoud et al., 2018; Titze et al., 2018). This approach is further evidence that using conductive metal particles around target samples facilitates serial SEM image acquisition, especially SBEM, which is readily affected by charging artifacts.

Increasing conductivity without influencing the contrast of the embedding medium can be achieved by using conductive materials composed of light elements. Carbon-based materials have relatively high conductivity, and for example, conductive tape covered by carbon nanotubes was used for imaging with ATUM and SEM (Kubota et al., 2018). Carbon black fillers have been used to modify the physical properties of plastics and polymers, such as electrical conductivity and material toughness, and therefore the addition of carbon black to embedding media may improve conductivity without affecting contrast (Yacubowicz et al., 1990; Chekanov et al., 1999; Novák et al., 2005; Domun et al., 2015).

One type of commercially available carbon black, called Ketjen black, reduces the resistance of base resins without altering mechanical stability (Kim et al., 2008). The reduction in resistance depends on the amount of the carbon added to the resin, but Ketjen black increases conductivity at relatively low concentrations (Connor et al., 1998; Chekanov et al., 1999; Balberg, 2002). A more structured carbon black, such as Ketjen black, forms larger agglomerates, which results in networks of conductive fillers with small gaps and improves the conductivity of non-conductive base materials even at lower concentrations (Balberg, 2002). Together, these studies suggest that Ketjen black is the most suitable carbon black for electron microscopy because it efficiently reduces resistance while maintaining mechanical stability.

**Figure 4:**
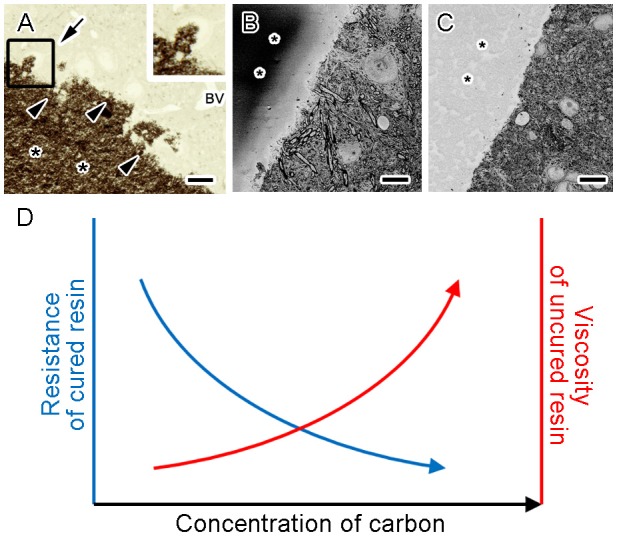
Higher concentrations of Ketjen black increased conductivity and viscosity of the conductive resin. A light microscope image of the section obtained from a mouse brain tissue embedded in the conductive resin shows dark granular aggregates of carbon (**A**, asterisks) in the vicinity of the tissue (**A**, arrowheads) but little penetration into the tissues (**A**, arrow). BV, blood vessel. Scanning electron microscope block-face images show abnormal contrast was prominent in resin without carbon black (**B**, asterisks) but eliminated in resin with Ketjen black (**C**, asterisks). The schematic graph shows resistance of the block decreased (**D**, blue arrow) and viscosity of uncured resin increased (**D**, red arrow) when conductive resin contained increasing concentrations of Ketjen black. Bars: 20 μm **(A)** or 10 μm **(B,C)**. Images **(A–C)** were adapted from Nguyen et al. (2016) with permission.

Indeed, conductive resin produced by Ketjen black is useful for imaging with SBEM under several different sample preparations (Figure 3C; Thai et al., 2016). Ketjen black particles are too large to enter cells and tissues, and therefore cannot penetrate deep inside tissues even when well dispersed in base resins and incubated with samples for a long time (Figure 4A). However, the addition of conductive materials in the resin substantially diminishes charging of the samples and resins for SBEM imaging (Figures 4B,C; Nguyen et al., 2016). In addition, embedding Ketjen black into resin ameliorates image deformation caused by insufficient sample conductivity, improves slicing quality and facilitates acquisition of serial images at higher resolution (Nguyen et al., 2016). Conductive resins based on carbon black fillers substantially reduce charging artifacts, result in better ultrastructural data and are applicable to various types of tissues in SBEM imaging (Nguyen et al., 2016, 2018; Thai et al., 2016, in press; Yoshimura et al., 2017; Saitoh et al., 2018; Sawada et al., 2018; Takeda et al., 2018).

## Future Perspectives of the Embedding Media for Volume Imaging

Although the currently available conductive resins have beneficial effects in volume imaging with SEM, there are several drawbacks in their usage. For example, the amount of the carbon black that can be added is limited partly by the increased viscosity of uncured resin (Lee, 1992; Nguyen et al., 2016). Addition of more Ketjen black into the resin results in further reduction of resistance, and also further increase in viscosity (Figure 4D), which impairs sample embedding. Therefore, the amount of Ketjen black that can be added to the resin is limited by the maximum viscosity acceptable for embedding. It is important to choose the concentration of Ketjen black where the resistance of the cured block and viscosity of the uncured resin are at acceptable levels. This issue might be partly addressed by selection of the base resins with lower viscosity. At the same time, selection of appropriate base resins and embedding media which will reduce deformations from electron irradiation facilitates better serial image acquisition (Kizilyaprak et al., 2015). Future studies might elucidate the optimal selection of the embedding media with acceptable viscosity and deformations, which would significantly facilitate production of conductive resins and acquisition of high quality data from biological specimens.

In addition, carbon-based resins in general require careful dispersion of the carbon powder during mixing with the base resin. Suboptimal dispersion impairs conductivity of the resins produced with the conductive fillers. Metallic filler materials would also have similar requirement of dispersion, and usage of premixed products which are commercially available reduced the burden of manual dispersion of the fillers (Wanner et al., 2016). Development and distribution of such premixed products would be preferred for the future conductive embedding media with conductive filler particles used for electron microscopic imaging.

Lastly, the reduced transparency or complete opacity of the samples applies not only to carbon-filled resins, but also to the other filler materials. These issues are attributable to the non-transparent properties of the filler materials added to the base resins. Although improvement in the conductivity of the base resin could not be achieved so far by addition of transparent and conductive ionic liquid (Nguyen et al., 2016), exploration and application of transparent conductive materials might lead to development of conductive embedding media which is preferred for the identification and orientation of the embedded samples without exposure.

## Concluding Remarks

During the past several years, there have been rapid methodological advancements for volume imaging of large biological specimens with SEM including increased options for staining, embedding and observation. Conductive materials are a unique option for better quality of images by reducing the charging of sample blocks in serial image acquisition with SEM, which is prone to charging artifacts. The available methods still have many limitations, and future studies involving the development and application of novel materials and a combination of available modifications may lead to better scale, quality, and throughput for the three-dimensional ultrastructural analyses of biological samples. These efforts will enable a deeper understanding of neural circuitry and provide the structural foundation for basic and higher brain functions.

## Author Contributions

All authors contributed to the writing and approved the final version of the manuscript.

## Conflict of Interest Statement

The authors declare that the research was conducted in the absence of any commercial or financial relationships that could be construed as a potential conflict of interest. The handling editor declared a shared affiliation, though no other collaboration, with several of the authors HN, TT and NO at time of review.
